# Protective Effect of Indole-3-Aldehyde in Murine COVID-19-Associated Pulmonary Aspergillosis

**DOI:** 10.3390/jof10070510

**Published:** 2024-07-22

**Authors:** Marilena Pariano, Anna Gidari, Claudia Stincardini, Sara Pierucci, Sabrina Bastianelli, Matteo Puccetti, Stefano Giovagnoli, Marina M. Bellet, Consuelo Fabi, Roberto Castronari, Cinzia Antognelli, Claudio Costantini, Maurizio Ricci, Daniela Francisci, Luigina Romani

**Affiliations:** 1 Department of Medicine and Surgery, University of Perugia, 06132 Perugia, Italy; marilena.pariano@gmail.com (M.P.); anna.gidari@studenti.unig.it (A.G.); claudiastincardini@gmail.com (C.S.); sara.pierucci@unipg.it (S.P.); sabrina.bastianelli@unipg.it (S.B.); marinamaria.bellet@unipg.it (M.M.B.); consuelofabi93@gmail.com (C.F.); roberto.castronari@unipg.it (R.C.); cinzia.antognelli@unipg.it (C.A.); claudio.costantini@unipg.it (C.C.); daniela.francisci@unipg.it (D.F.); 2 Department of Pharmaceutical Sciences, University of Perugia, 06132 Perugia, Italy; matteo.puccetti@gmail.com (M.P.); stefano.giovagnoli@unipg.it (S.G.); maurizio.ricci@unipg.it (M.R.)

**Keywords:** CAPA, aspergillosis, SARS-CoV-2, indole-3-aldehyde

## Abstract

*Aspergillus fumigatus* is an environmental fungus recently included in the fungal high-priority pathogens by the World Health Organization. While immunodeficiency and/or pre-existing lung damage represent a well-recognized fertile ground for fungal growth, it is increasingly being recognized that severe viral infections may similarly favor *A. fumigatus* colonization and infection, as recently experienced in the Coronavirus disease 2019 (COVID-19) pandemic. Herein, in a murine model of COVID-19-associated pulmonary aspergillosis (CAPA), obtained by the concomitant exposure to the Severe Acute Respiratory Syndrome Coronavirus 2 (SARS-CoV-2) Spike protein and *A. fumigatus* conidia, we found that the microbial compound indole-3-aldehyde (3-IAld) was able to ameliorate CAPA by working at multiple levels during viral infection and fungal superinfection, including epithelial barrier protection, promotion of antiviral responses, and limiting viral replication. As a consequence, 3-IAld limited the pathogenic sequelae of fungal superinfection as revealed by the controlled fungal burden and restrained inflammatory pathology. These results point to indole compounds as potential agents to prevent CAPA.

## 1. Introduction

Fungal diseases are an important cause of morbidity and mortality in humans. In recognition of their impact on human health, the World Health Organization has recently elaborated a fungal priority pathogens list to guide research and policy interventions [1]. In an attempt to prioritize fungal pathogens, three categories were defined, i.e., critical-, high-, and medium-priority groups. Within the former, the mold *Aspergillus fumigatus* and the yeasts *Cryptococcus neoformans*, *Candida albicans*, and *C. auris* were included [1]. Current estimates show that over 6.5 million people annually are affected by a severe fungal disease, with over 3.75 million deaths, 68% of which are directly attributable to the fungal disease [2]. Higher incidence was reported for invasive aspergillosis, chronic pulmonary aspergillosis, and candidemia and invasive candidiasis, with invasive aspergillosis associated with the higher crude mortality [2].

*A. fumigatus* is an environmental saprotrophic fungus whose life cycle is characterized by the production of asexual spores, or conidia, that are efficiently dispersed in the environment [3]. Humans are constantly exposed to *A. fumigatus* conidia that enter the upper respiratory tract to reach the alveoli. In the immunocompetent host, the conidia are efficiently removed by mechanical and immunological mechanisms, based on mucociliary clearance and removal by alveolar macrophages, respectively [3]. If clearance fails, *A. fumigatus* conidia can germinate and initiate hyphal growth. The clinical picture of *A. fumigatus* colonization and/or infection is tailored to the host phenotype. Immunocompromised individuals, such as those undergoing hematopoietic stem cell transplantation or in induction chemotherapy for leukemia, may develop a life-threatening invasive aspergillosis, while aspergilloma or chronic pulmonary aspergillosis may affect patients with pre-existing lung damage. *A. fumigatus* may also be the cause of asthmatic reactions, such as in chronic bronchopulmonary aspergillosis [3].

The recent Coronavirus disease 2019 (COVID-19) pandemic caused by the Severe Acute Respiratory Syndrome Coronavirus 2 (SARS-CoV-2) has recently expanded the clinical scenarios of *A. fumigatus* infection to include COVID-19-associated pulmonary aspergillosis (CAPA) [4,5,6,7]. A multicenter study in France reported a 15% prevalence of CAPA in critically ill patients requiring invasive mechanical ventilation during the first wave [8], and a 5.1% prevalence in the Delta and Omicron waves, which rose to 9.1% among patients who required invasive mechanical ventilation [9]. Along with influenza-associated pulmonary aspergillosis, CAPA represents a form of viral-associated pulmonary aspergillosis in which a severe viral infection, likely by disrupting epithelial integrity and inducing a severe inflammatory response in critical patients, predisposes to the development of invasive fungal infection [7]. Therefore, protection against mucosal barrier impairment might be crucial to prevent fungal superinfection after severe viral disease.

The commensal microbes that colonize the bodily surfaces exposed to the external environment, collectively known as microbiota, and their metabolites, such as short-chain fatty acids (SCFAs) and tryptophan (Trp)-derived indolic compounds, are increasingly being recognized for their role in maintaining mucosal homeostasis [10]. One such compound, indole-3-aldehyde (3-IAld), was identified by our group as a product of Lactobacilli under conditions of unrestricted Trp availability with the ability to provide antifungal resistance and protection against mucosal damage [11,12]. The activities associated with 3-IAld have since expanded to confirm its potent role in promoting epithelial barrier function, immune homeostasis, and colonization resistance at mucosal sites [13].

Based on these premises, we herein established a murine model of CAPA and explored the potential therapeutic activity of 3-IAld in this model. Our results show that 3-IAld exerted a multiplicity of effects in CAPA, from nasal barrier protection to the inhibition of viral replication and subsequent *Aspergillus* superinfection.

## 2. Materials and Methods

### 2.1. Mice, Infections, and Treatments

Male BALB/c mice, 8–10 weeks old, were purchased from Charles River Laboratories (Calco, Italy). To establish a murine model of CAPA, mice were anesthetized via intraperitoneal injection with Penthotal, (50 mg/kg) before intratracheally instillation of 15 μg/mice of SARS-CoV-2 recombinant wild-type (WT) Spike protein (SPwt) (ECD, His & Flag Tag, Genescript, Z03481) or Delta (Δ) Spike protein (SPΔ) (RBD, L452R, T478K, Avi & His Tag, Genescript, Z03613) to mimic SARS-CoV-2 infection. Contextually, 1.5 × 10^8^ *A. fumigatus* (Af293) resting conidia/50 μL of saline were administered to mimic fungal superinfection. The dose of the Spike protein was derived from a previously established model of SARS-CoV-2 infection [14] while the infectious dose of the fungus was chosen to obtain measurable fungal growth at 3 dpi as per protocol. Four mice were used in each experiment. Quantification of fungal growth was performed as described [7]. Briefly, fungal growth was expressed as colony-forming units (log_10_ CFUs), obtained by serially diluting lung homogenates on Sabouraud agar plates incubated at 35 °C for 24 h. Bronchoalveolar lavage (BAL) was performed by cannulating the trachea and washing the airways with 3 × 0.5 mL of PBS to collect the BAL fluid. For differential BAL fluid cell counts, cytospin preparations were made and stained with May–Grünwald Giemsa reagents (Sigma–Aldrich, St. Louis, MO, USA). For histology, lungs were removed and immediately fixed in 10% neutral buffered formalin (Bio-Optica Milano Spa, Milan, Italy) for 24 h. The fixed organs were dehydrated, embedded in paraffin, sectioned into 3–4 μm, and stained with Periodic Acid-Schiff reagent (Abcam, Cambridge, UK). 3-IAld (Sigma-Aldrich, Milan, Italy) was encapsulated into microparticles (2:1 Eudragit^®^ S100:L100 ratio with 30% *w*/*w* ETHOCEL std. 7 (Dow Chemical Company, Milan, Italy) in ethanol), as detailed [15]. It was given orally at a dose of 18 mg/kg in 200 µL of PBS, a day before and a day post-infection and SP administration. Mice were sacrificed at 3 days post-infection. Infections were performed under isoflurane anesthesia, and all efforts were made to minimize suffering. Mouse experiments were performed according to Italian Approved Animal Welfare Authorization 360/2015-PR and Legislative Decree 26/2014, regarding the animal license obtained by the Italian Ministry of Health lasting for 5 years (2015–2020).

### 2.2. SiRNA Design and Delivery

Predesigned SiRNA against *Ace2* (mm.Ri.Ace2.13.1) were purchased from Integrated DNA Technologies (IDT) (TEMA Ricerca Srl, Castenaso, Bologna, Italy). For in vivo experiments, each mouse received intranasal administration of 10 μg/kg unmodified SiRNA or equivalent dose of nonspecific control SiRNA duplex (scramble) in a volume of 20 μL of duplex buffer (IDT). Intranasal SiRNA was given the day before and the day after the infection.

### 2.3. TUNEL Staining

Sections were deparaffinized, rehydrated, and treated with 0.1 M citrate buffer, pH 6.0, for 20 min in a water bath at 95 °C, washed, and fixed in 4% buffered paraformaldehyde, pH 7.3, for 36 h. The sections were then washed and blocked in 0.1 M Tris/HCl buffer, pH 7.5, and supplemented with 3% bovine serum albumin and 20% FCS. The slides were then incubated with fluorescein-coupled dUTP and TUNEL enzyme (Roche Diagnostics SpA, Monza, Italy) in the presence of terminal deoxynucleotidyltransferase. The samples were then washed with PBS, incubated for 10 min at 70 °C to remove unspecific binding. The sections were mounted and analyzed by fluorescent microscopy using a 20× objective.

### 2.4. Real-Time PCR

Real-time PCR was performed using the CFX96 Touch Real-Time PCR detection system and iTaq Universal SYBR Green Supermix (Bio-Rad Laboratories, Inc., Hercules, CA, USA). Organs were lysed and total RNA was extracted using TRIzol Reagent (Thermo Fisher Scientific, Waltham, MA, USA) and reverse transcribed with PrimeScript RT Reagent Kit with gDNA Eraser (Takara Bio. Inc., Kusatsu, Japan), according to the manufacturer’s directions. Amplification efficiencies were validated and normalized against β-actin. The thermal profile for SYBR Green real-time PCR was at 95 °C for 3 min, followed by 40 cycles of denaturation for 30 s at 95 °C, and an annealing/extension step of 30 s at 60 °C. Each data point was examined for integrity by analysis of the amplification plot. The mRNA-normalized data were expressed as relative mRNA levels with respect to the control. The primers are listed in Table 1.

### 2.5. Cytokine Determination by ELISA

Cytokine content was determined in lung homogenates by using specific ELISA kits according to the manufacturer’s instructions (BioLegend Inc., San Diego, CA, USA, cod. 432616).

### 2.6. Cells, Infection, and Treatments

Vero (ATCC-CCl-81) E6 monolayer cells cultured in 96-well plates (3 × 10^4^ cells/well) were exposed to 10 μM of unformulated 3-IAld (Sigma Aldrich) or 50 μM of the natural phytochemical [16], indole-3-carbinole (I3C) (Sigma Aldrich), or 10 μM of Remdesivir (Veklury^®^, Gilead, Foster City, CA, USA) either 1 h before (prophylactic protocol) or 1 h after (therapeutic protocol) the infection with the SARS-CoV-2-B.1.1.7 variant of concern at a multiplicity of infection (MOI), defined as the ratio of agents (virus) to infection targets (cell), =0.001, using ATCC-formulated Eagle’s Minimum Essential Medium (EMEM) supplemented with heat-inactivated 2% FBS, 2 mM L-glutamine, and 1% penicillin–streptomycin. The B.1.1.7 SARS-CoV-2 strain has been isolated from a nasopharyngeal swab of a symptomatic patient. The virus isolation was performed in the Biosafety Level 3 (BSL3) Virology laboratory at “Santa Maria della Misericordia Hospital”, Perugia, Italy, as previously described [17]. The compounds were dissolved to 10 mM in DMSO and then diluted in culture medium. DMSO (1 and 0.01% (*v*/*v*)) was used as the control.

RPMI 2650 (ATCC-CCL-30) cells were maintained in MEM supplemented with 10% fetal bovine serum (FBS), 1% sodium pyruvate, GlutaMAX 4 mM, and 0.2% Non-Essential Amino Acids Solution. The cells were maintained at 37 °C in a humidified atmosphere containing 5% CO_2_. Cells were seeded on 6-well tissue culture plates and infected with the SARS-CoV-2 variant at an MOI = 0.001 after pre-treatment for 1 h with 10 μM of 3-IAld or 50 μM of I3C. Cells were assessed for viability and gene expression after 24 h of viral exposure. Experiments and sample processing were conducted in the BSL3 laboratory.

### 2.7. Plaque Reduction Assay

Supernatants’ viral titer was determined by plaque assay as previously described [18] with some modifications. Vero E6 cells (600,000 cells/well) were seeded in a 6-well plate and incubated at 37 °C with 5% CO_2_ for 24 h in EMEM supplemented with heat-inactivated 2% FBS and 2 mM L-glutamine. After incubation, the medium was removed and cells were infected with 500 µL of ten-fold serial dilution of the supernatants, rocking the plates every fifteen minutes. In the meanwhile, the overlay medium (complete medium with Agar 0.1%) was prepared and maintained in a 50 °C water bath. After 1 h of infection, the overlay medium (2 mL) was poured into each well and the plates were incubated for 3 days at 37 °C. Finally, the overlay was discarded, cells were fixed for 30 min with 4% formalin, and stained with 0.5% crystal violet. Viral titer was determined as plaque-forming units per ml (PFU/mL), considering wells with plaques ranging from 2 to 100. For each pool of supernatants, plaque reduction assay was performed in duplicate. Experiments were conducted in the BSL3 laboratory.

### 2.8. Cytopathic Effect Inhibition Assay

Monolayers of Vero E6 cells growing in 96-well plates (3 × 10^4^ cells/well) were exposed to 10 μM of 3-IAld or 50 μM of I3C in DMSO, 1 h before or 1 h after SARS-CoV-2 infection at an MOI = 0.001 using EMEM supplemented with heat-inactivated 2% FBS and 2 mM L-glutamine. DMSO (0.5 and 0.05% (*v*/*v*)) was used as the control. Cell viability was measured 72 h later by a standard crystal violet staining assay, measuring the optical density (OD) at 595 nm. The extent of in vitro inhibition of SARS-CoV-2-driven cell damage was expressed as percentage of surviving cells.

### 2.9. Statistical Analysis

GraphPad Prism software 6.01 (GraphPad Software, San Diego, CA, USA) was used to determine the statistical significance. Significance was defined as *p* < 0.05. Data are expressed as mean ± SD. Statistical significance was calculated by one-way ANOVA (Tukey’s or Bonferroni’s post hoc test) for multiple comparisons.

## 3. Results

### 3.1. SARS-CoV-2 Spike Protein Worsens Aspergillus Infection in a Murine Model of CAPA

**Figure 1:**
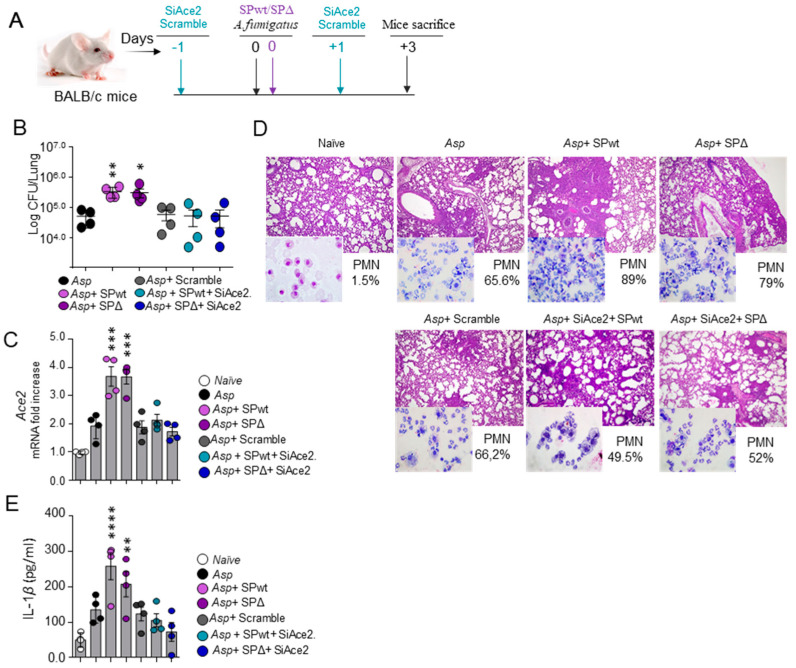
**SARS-CoV-2 Spike protein worsens *Aspergillus* infection in murine CAPA**. BALB/c mice were intratracheally infected with *Aspergillus fumigatus* conidia and treated with the Spike protein from the wild-type (SPwt) or the Delta (SPΔ) variant and siRNA of *Ace2* (SiAce2) as indicated in (**A**). Mice were evaluated for (**B**) fungal growth (log_10_ CFU, mean ± SEM); (**C**) expression of *Ace2* in the lung by RT-PCR; (**D**) lung histopathology and neutrophil recruitment (% in the BAL, in the inset); and (**E**) IL-1β production (by ELISA). Images were taken with a high-resolution microscope (Olympus BX51), 20× magnification (scale bars, 200 μm). Data are presented as mean ± SD of one representative out of three independent experiments. * *p* < 0.05, ** *p* < 0.01, *** *p* < 0.001, **** *p* < 0.0001, *Aspergillus*-infected mice treated with the SPwt and/or SPΔ, with or without SiAce2 vs. *Aspergillus*-infected mice, one-way ANOVA, Bonferroni *post hoc* test.

In order to establish a model of CAPA, we resorted to a surrogate model of SARS-CoV-2 infection [14], in which BALB/c mice are concomitantly exposed to *A. fumigatus* conidia and the SARS-CoV-2 Spike protein, either from the WT virus or the Δ variant (Figure 1A). We found that the concomitant treatment with either form of SARS-CoV-2 Spike protein significantly increased the fungal burden (Figure 1B) and worsened the lung histopathology while increasing neutrophil infiltration in the BAL (Figure 1D). Treatments also increased the levels of the SARS-CoV-2 Spike protein receptor Angiotensin-converting enzyme 2 (*Ace2*) expression in the lung (Figure 1C) and of IL-1β in the lung homogenate (Figure 1E). Blocking ACE2 by the local delivery of SiRNA against *Ace2* during the concomitant exposure to the Spike protein and *Aspergillus* conidia (Figure 1C) prevented the increased fungal burden and the ensuing inflammatory response (Figure 1D,E), indicating that the binding of the SARS-CoV-2 Spike protein to its ACE2 receptor is mediating the worsening effect on the *A. fumigatus* infection.

These results indicate that the concomitant treatment with SARS-CoV-2 Spike protein and *A. fumigatus* conidia may represent a valuable model of CAPA whereby viral challenge increases the susceptibility to fungal disease.

### 3.2. 3-IAld Protects against CAPA

**Figure 2:**
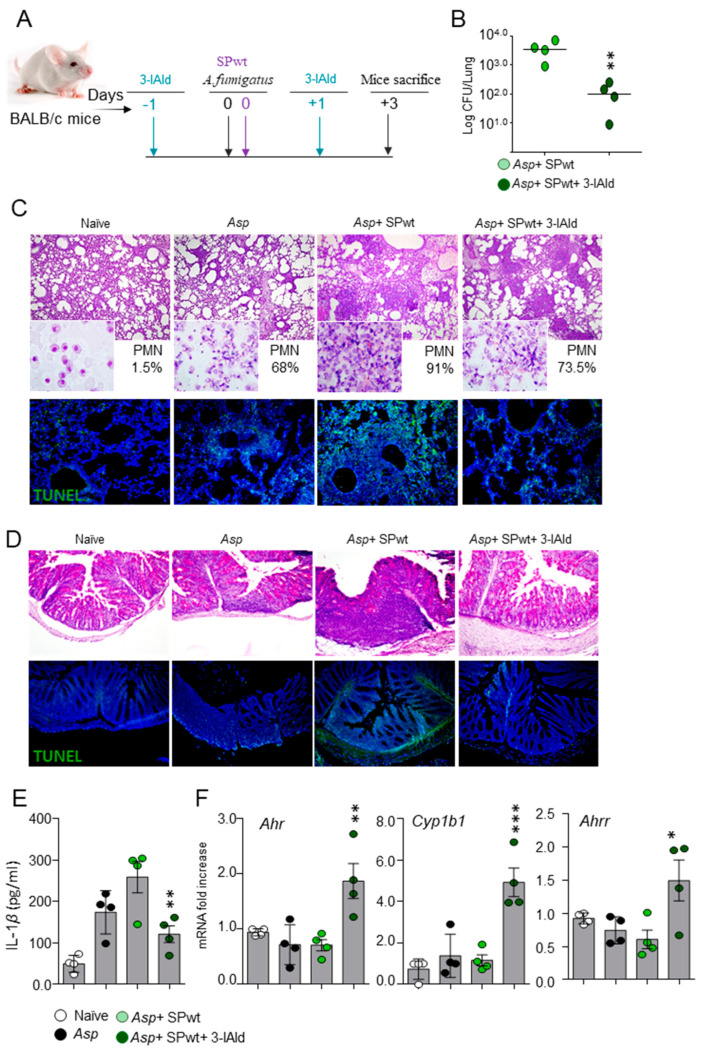
**3-IAld protects against CAPA**. BALB/c mice were intratracheally infected with *Aspergillus fumigatus* conidia, exposed to the wild-type Spike protein (SPwt), and treated with 3-IAld administered orally at a dose of 18 mg/kg, a day before and a day after the infection, as illustrated in (**A**). Mice were evaluated for (**B**) fungal growth (log_10_ CFU, mean ± SEM); (**C**,**D**) lung (neutrophil recruitment in the BAL, in the inset) and intestine histopathology, and TUNEL staining; (**E**) IL-1β production (ELISA) in lung homogenates; and (**F**) expression of *Ahr*, *Cyp1b1*, and *Ahrr* in the lung by RT-PCR. Images were taken with a high-resolution microscope (Olympus BX51), 20× magnification (scale bars, 200 μm). Data are presented as mean ± SD of one representative out of three independent experiments. * *p* < 0.05, ** *p* < 0.01, *** *p* < 0.001, *Aspergillus*-infected mice treated with SPwt and 3-IAld vs. *Aspergillus*-infected mice treated only with SPwt. One-way ANOVA, Bonferroni *post hoc* test.

Since the SPwt and the SPΔ variant of the SARS-CoV-2 Spike protein produced overlapping effects in our model of CAPA, subsequent experiments were performed only with the SPwt. In order to explore the potential therapeutic application of 3-IAld, BALB/c mice were exposed to *A. fumigatus* conidia and SPwt as above and treated with 3-IAld, formulated in microparticles for small intestine local delivery [15], 1 day before and 1 day after the viral and fungal challenge (Figure 2A). Mice were sacrificed 3 days later and analyzed for fungal burden, lung histopathology, and IL-1β levels. We found that treatment with 3-IAld significantly reduced the fungal burden (Figure 2B), improved the lung inflammatory pathology (Figure 2C), reduced the number of TUNEL-positive apoptotic epithelial cells (Figure 2C), and the IL-1β levels in lung homogenates (Figure 2E). These results indicate that 3-IAld protects from inflammatory pathology and epithelial damage in fungal pneumonia.

### 3.3. 3-IAld Restores Mucosal Homeostasis in CAPA via the Aryl Hydrocarbon Receptor

Since 3-IAld was locally administered in the intestine, where it is known to exert barrier-enhancing and anti-inflammatory effects [13], we also evaluated signs of inflammatory pathology in the colon. As expected, administration of 3-IAld prevented colon damage and reduced the number of TUNEL-positive apoptotic cells in CAPA (Figure 2D), indicating that 3-IAld is able to afford epithelia barrier protection locally and at distal sites. In line with 3-IAld being a ligand of the Aryl Hydrocarbon Receptor (AhR) [19], a ligand-activated transcription factor involved in the regulation of multiple physiological functions, including the regulation of the mucosal immune homeostasis [20], treatment with 3-IAld resulted in the up-regulation of the AhR target genes [21], *Cyp1b1*, and the AhR repressor (*Ahrr*) (Figure 2F) in the lung, as already observed in the intestinal tract [15].

Collectively, these results indicate that 3-IAld is able to protect against the epithelial damage and inflammatory effects caused by the combined viral and fungal challenge in murine CAPA.

### 3.4. 3-IAld Counteracts the SARS-CoV-2 Immunomodulatory Effects in Nasal Epithelial Cells

**Figure 3:**
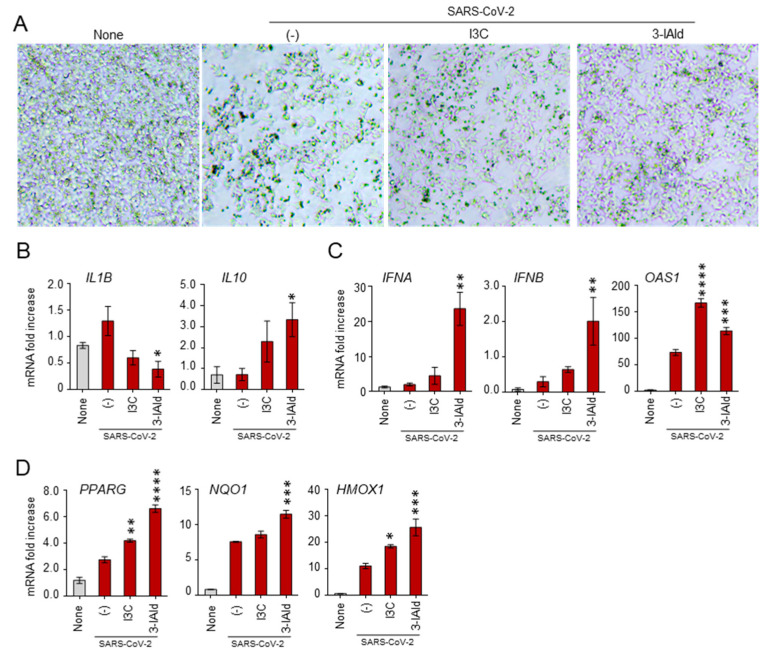
**3-IAld counteracts the SARS-CoV-2 immunomodulatory effects in nasal epithelial cells**. RPMI cells were pre-treated for 1 h with 10 μM of 3-IAld in DMSO or 50 μM of I3C, exposed to the SARS-CoV-2, B.1.1.7 variant of concern, and evaluated for (**A**) morphology and expression of (**B**) *IL-1β* and *IL-10* cytokines genes (**C**), antiviral type I IFNs and *OAS1* genes, and (**D**) *PPARG*, *NQO1*, and *HMOX-1* oxidative stress genes. Data are presented as mean ± SD of two independent experiments. * *p* < 0.05, ** *p* < 0.01, *** *p* < 0.001, **** *p* < 0.0001, treated with I3C and 3-IAld vs. untreated virus-exposed cells, one-way ANOVA, Bonferroni *post hoc* test.

While *A. fumigatus* conidia reach the lung alveoli owing to their small size [3], SARS-CoV-2 first interacts with ACE2 in the nasal mucosa, and only subsequently translocates to the lungs, likely by aspiration [22]. Therefore, the nasal mucosa represents a critical barrier against SARS-CoV-2 infection and its integrity should be preserved to protect against viral entry [23]. Based on these premises, we have resorted to RPMI cells, a human nasal epithelial cell line, infected with SARS-CoV-2 (B.1.1.7 variant of concern) to evaluate the potential effect of 3-IAld in the modulation of genes involved in the antiviral and inflammatory response. As a positive control, we used I3C, known for its antiviral activity including against SARS-CoV-2 [24,25,26]. As shown in Figure 3A,B, challenge with SARS-CoV-2 altered the number and morphology of RPMI cells while inducing the production of the pro-inflammatory cytokine IL-1 β. Interestingly, the effects induced by viral exposure were partially rescued by I3C and, even more, by 3-IAld. In particular, both I3C and 3-IAld reverted the viral-induced pro-inflammatory profile by reducing IL-1β production and increasing IL-10 (Figure 3B), while potentiating the expression of the antiviral type I IFNs and 2′−5′-oligoadenylate synthetase (*OAS1*) genes (Figure 3C). In addition, both I3C and, even more, 3-IAld increased the expression of genes involved in the response to oxidative stress, such as Peroxisome Proliferator-Activated Receptor Gamma (*PPARG*), NAD(P)H Quinone Dehydrogenase 1 (*NQO1*), and Heme Oxygenase 1 (*HMOX-1*) (Figure 3D).

Altogether, these results indicate that 3-IAld is able to modulate the antiviral, inflammatory, and oxidative stress responses in the nasal epithelium, thus likely preventing the initial steps of SARS-CoV-2 infection.

### 3.5. 3-IAld Exerts Direct Antiviral Effects In Vitro

**Figure 4:**
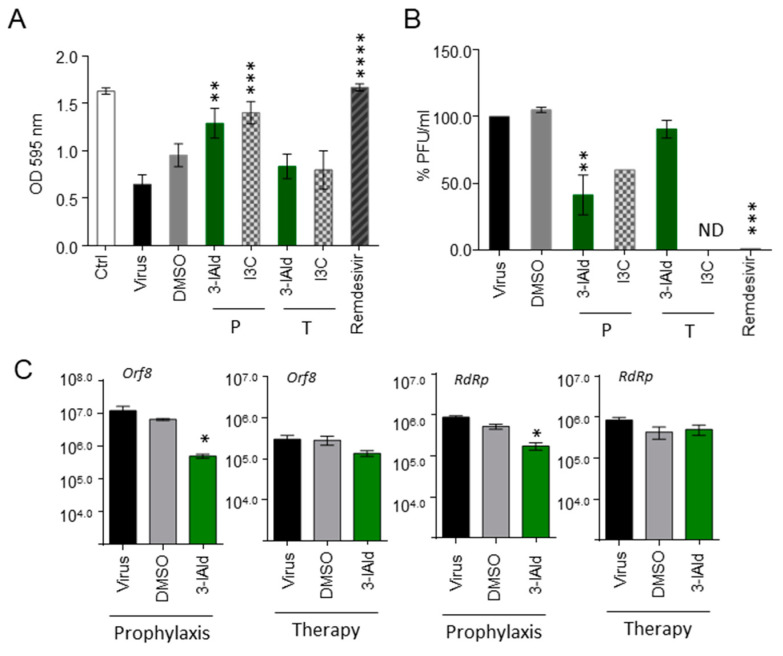
**3-IAld exerts direct antiviral effects in vitro**. Vero E6 cells were infected with the SARS-CoV-2 strain and exposed to 10 μM of 3-IAld, 50 μM of I3C in DMSO, or 10 μM of Remdesivir, either 1 h before (P, prophylaxis) or 1 h after (T, therapy) SARS-CoV-2 infection. The compounds were dissolved to 10 mM in DMSO and then diluted in culture medium. DMSO (1 and 0.01% (*v*/*v*)) was used as control. Cells were assessed for (**A**) viability by the standard crystal violet staining assay, measuring the optical density (OD) at 595 nm; (**B**) viral titer as plaque-forming units per ml; (**C**) expression of Orf8 and RdRp by RT-PCR. Data are presented as mean ± SD of two independent experiments. * *p* < 0.05, ** *p* < 0.01, *** *p* < 0.001, **** *p* < 0.0001, treated with I3C and 3-IAld vs. untreated virus-exposed cells, one-way ANOVA, Bonferroni post hoc test. ND, not determined.

Given the antiviral activity of indole derivatives containing an indole core framework [27], we tested whether 3-IAld could also directly have antiviral effects by inhibiting SARS-CoV-2 replication. For this reason, we resorted to VeroE6 cells, kidney epithelial cells extracted from an African green monkey, infected with SARS-CoV-2 in the presence or not of 3-IAld or I3C, each supplied either before or after viral exposure at a concentration found to be optimal ([25] and Supplementary Figure S1). As a positive control, the broad-spectrum antiviral drug Remdesivir was used in parallel. As shown in Figure 4A, both 3-IAld and I3C significantly inhibited SARS-CoV-2 replication and VeroE6 cell death in the prophylactic, but not therapeutic, protocol. In line with these observations, a prophylactic, but not therapeutic, administration of 3-IAld reduced the levels of the Open Reading Frame 8 (*Orf8*) and RNA-dependent RNA polymerase (*RdRp*) viral genes (Figure 4C).

Collectively, these results indicate that 3-IAld is potentially able to counteract the SARS-CoV-2 infection by modulating the expression of genes involved in mucosal protection and immune response, as well as by directly inhibiting viral replication.

## 4. Discussion

The results presented in this study demonstrate that the microbial-derived product 3-IAld is able to limit the pathogenic consequences of SARS-CoV-2 infection and subsequent *Aspergillus* superinfection by working at multiple levels. Indeed, 3-IAld was able to limit viral replication by acting directly on the virus and to switch the host immune system from a pathogenic pro-inflammatory state to a protective antiviral response. These activities likely occur at the nasal mucosa, which represents the entry site for the virus before translocation to the lower respiratory tract. Moreover, and likely, as a consequence, 3-IAld limited the pathogenic sequelae of fungal superinfection as revealed by the controlled fungal burden and restrained inflammatory pathology in the lungs.

These results confirm and extend previous findings on the role of indole derivatives containing an indole core framework to prevent and/or treat viral infections [27]. For example, a small-molecule compound with an indole moiety was found to inhibit the main protease of SARS-CoV-2 and to block virus replication [28]. Even more, arbidol, an indole derivative that inhibits virus entry, membrane fusion, and viral release [29,30], is approved as a prophylactic and therapeutic agent to treat influenza and other acute respiratory viral infections in Russia and China [31]. Of interest, the anti-influenza virus activity of arbidol was comparable to that of 3-IAld [32]. Studies have also shown that arbidol significantly contributed to both clinical and laboratory improvements in COVID-19 patients [33,34], arguing for a wide-ranging antiviral activity these compounds may have.

Our results indicate that 3-IAld works better in a prophylactic rather than therapeutic protocol. The same observation has been reported for I3C, whose antiviral activity during SARS-CoV-2 infection in Vero E6 cells was better observed in a pre-treatment protocol compared to co- and post-treatment protocols [25]. At least in the case of 3-IAld, the reason for this result may be linked to the mechanism of action. For instance, it has been previously shown that ligands of AhR inhibited SARS-CoV-2 infection of Vero E6 cells by reducing the expression of ACE2 [35]. Should 3-IAld, a ligand of AhR, work by means of a similar mechanism, this might explain why administration before SARS-CoV-2 infection has optimal efficacy because it would interfere with the initial steps of viral entry.

It is increasingly being recognized that 3-IAld can engage a variety of cellular and molecular mechanisms in the different settings in which it has been studied [13], some of which may be working in the protection against SARS-CoV-2 infection and subsequent *Aspergillus* superinfection. For instance, it has been shown that small-molecule mast cell (MC) activators may enhance immunity in the nasal mucosa and may be used as nasal vaccine adjuvants [36]. Interestingly, 3-IAld is able to modulate the Trp metabolism in MCs and protect against autoimmune manifestations in multiple sclerosis [19], raising the interesting hypothesis that 3-IAld may be used to engage MCs in the nasal mucosa in vaccination strategies against COVID-19. 3-IAld could also modulate the composition and function of the microbiome. Indeed, we have previously shown that 3-IAld increases the relative abundance of pectin-degrading and sugar-degrading anaerobic bacteria resulting in increased levels of fecal SCFA and likely hydrogen sulfide (H_2_S), an end-product of the bacterial fermentation of pectin [37]. Of note, it has been proposed that H_2_S may target SARS-CoV-2 cell entry, replication, and induction of inflammatory response [38]. Whether 3-IAld may induce H_2_S with functional activity in the nose remains to be addressed.

The potential mechanisms engaged by 3-IAld depend on AhR activation, whose role in COVID-19 is controversial. Some studies have found that AhR is negatively correlated with SARS-CoV-2 infection and the outcome. Indeed, it was shown that AhR is activated by an IFNβ or IFNγ/indoleamine 2,3-dioxygenase (IDO)1/kynurenine (kyn) pathway in alveolar epithelial cells to increase the expression of mucins and induce hypoxia [39]. In addition, AhR activation promoted SARS-CoV-2 infection and replication, limited type I IFN signaling, and up-regulated the expression of ACE2, thus acting as a proviral factor [40,41] whose inhibition was suggested to be used as a therapeutic approach in COVID-19. At variance with these studies, a repurposing screen identified Phortress, an AhR-activating ligand, as an antiviral candidate for both an Alpha- and a Beta-coronavirus [42]. Similarly, as previously mentioned, AhR activation decreased the ACE2 expression and SARS-CoV-2 infection of Vero E6 cells [35]. This is in line with the antiviral activity of AhR agonists against Influenza A virus [43] and the protection against Acute Respiratory Distress Syndrome [44]. We have similarly found that AhR activation by 3-IAld was associated with type I IFN production and interference with SARS-CoV-2 replication in nasal cells and VeroE6 cells.

The different ligand- and tissue-specific activities of AhR [45] may account for these discrepancies. For instance, it was shown that 3-IAld could limit graft-versus-host disease (GvHD) by activating type I IFN signaling [46], consistent with the notion that not only the diversity of the ligands, but also the context in which AhR is activated may direct downstream signaling pathways. An inflammatory GvHD may redirect AhR activation towards type I IFN signaling while an antiviral background response may instead promote an AhR-mediated inflammatory signaling, thus making AhR a regulatory node to balance between the two responses. Tissue-specific differences in the expression and activation of AhR, and of AhR ligands as well, in the lower and higher respiratory tract may also be involved. For instance, scRNA studies in the respiratory tract [47,48,49] have shown that IDO1 is most expressed in the nose and belongs to the immune genes over-represented in the top ACE2-correlated genes [49]. In agreement with these studies, our results on Trp metabolites in hematological patients [50,51] indicate that the nose had higher levels of kyn compared with the pharynx [52]. This might suggest that in a background of high IDO1 activity, such as in the nose, AhR may be more responsive to the action of different ligands compared to the lungs where the levels of kyn are lower. Thus, at variance with the nose, the increased kyn levels in the lung upon viral-induced IFN signaling may activate AhR to promote a pro-inflammatory response. Should such a scenario prove to be plausible, AhR antagonists might prove more effective for local targeting into the lungs rather than for constraining AhR activity across the entire respiratory tree.

In conclusion, our results shed new light on the potential role of AhR in COVID-19 by virtue of its promiscuity of ligands and activities, and specifically put forward the hypothesis that microbial compounds, such as 3-IAld, owing to their multiplicity of activities and favorable safety profile [13], may be used as prophylactic agents in the protection against viral colonization and infection, and subsequent fungal superinfection.

## Figures and Tables

**Table 1:** List of primers.

**Murine Primers**	
*β-actin* *(Beta-Actin)*	forward AGCCATGTACGTAGCCATCC reverse CTCTCAGCTGTGGTGGTGAA
*Ace2* *(Angiotensin-Converting Enzyme 2)*	forward TCCATT-GGTCTTCTGCCATCC reverse AACGATCTCCCGCTTCATCTC
*Ahr* *(Aryl Hydrocarbon Receptor)*	forward TCCATCCTGGAAATTCGAACC reverse TCTTCATCCGTCAGTGGTCTC
*Ahrr* *(Aryl Hydrocarbon Receptor Repressor)*	forward AGAGGGTTCCCCGTGCAG reverse ACTCACCACCAGAGCGAAGC
*Cyp1b1* *(Cytochrome P450 Family 1 Subfam.B Member 1)*	forward TTCTCCAGCTTTTTGCCTGT reverse TAATGAAGCCGTCCTTGTCC
**Human Primers**	
*β-actin* *(Beta-Actin)*	forward CACTCTTCCAGCCTTCCTTCC reverse ACAGCACTGTGTTGGCGTAC
*IL1B* *(Interleukin 1 Beta)*	forward AAGCTCCTGTGGCAATTGAA reverse TCCTCCTTCTGGAACTGCTG
*IL10* *(Interleukin 10)*	forward CCTGCCTAACATGCTTCGAGA reverse TCTTGGTTCTCAGCTTGGGG
*IFNA1* *(Interferon Alpha 1)*	forward ACCCACAGCCTGGATAACAG reverse ACTGGTTGCCATCAAACTCC
*IFNB1* *(Interferon Beta 1)*	forward AGTAGGCGACACTGTTCGTG reverse GCCTCCCATTCAATTGCCAC
*OAS1* *(2′-5′-Oligoadenylate Synthetase 1)*	forward GAGACCCAAAGGGTTGGAGG reverse TCATCGTCTGCACTGTTGCT
*PPARG* *(Peroxisome Proliferator-Activated Receptor Gamma)*	forward TCGAGGACACCGGAGAGG reverse CACGGAGCTGATCCCAAAGT
*NQO1* *(NAD(P)H Quinone Dehydrogenase 1)*	forward GGTTTGGAGTCCCTGCCATT reverse ACCAGTGGTGATGGAAAGCA
*HMOX1* *(Heme Oxygenase 1)*	forward TGACCCATGACACCAAGGAC reverse AGTGTAAGGACCCATCGGAGA.
**Viral Primers**	
*Orf8* *(Open Reading Frame 8)*	forward GGTGCTGACTGAGAGCAATAA reverse CACATTAGAGCCGGTTGAGTAG
*RdRp* *(RNA-dependent RNA polymerase)*	Forward ACGCTCAAAGCTACTGAGGAGAC reverse GGTCTAGGTTTACCAACTTCCC

## Data Availability

Dataset available on request from the authors.

## Supplementary Materials

The following supporting information can be downloaded at: https://www.mdpi.com/article/10.3390/jof10070510/s1, Figure S1: 3-IAld exerts direct antiviral effects in vitro.
