# Supplementary material for: Protective Effect of Indole-3-Aldehyde in Murine COVID-19-Associated Pulmonary Aspergillosis
Source: J Fungi (Basel). 2024 Jul 22;10(7):510. doi: 10.3390/jof10070510 (PMC11278170; doi:10.3390/jof10070510)
Supplement: Supplementary file 1 [file jof-10-00510-s001.zip › jof-3070924-supplementary.pdf]

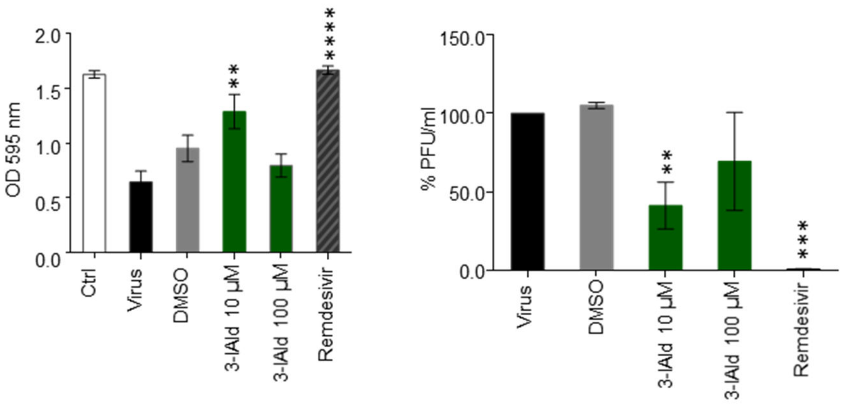

Supplementary Figure S1. 3-IAld exerts direct antiviral effects in vitro. Vero E6 cells were infected with the SARS-CoV-2 strain and exposed to 10 and 100 μM of 3-IAld in DMSO or 10 μM of Remdesivir, 1h before SARS-CoV-2 infection. The compounds were dissolved to 10 mM in DMSO and then diluted in culture medium. DMSO (1 and 0.01% (v/v)) was used as control. Cells were assessed for (A) viability by the standard crystal violet staining assay, measuring the optical density (OD) at 595 nm; (B) viral titer as plaque-forming units per ml. Data are presented as mean ± SD of two independent experiments. ** $p < 0.01$, *** $p < 0.001$, **** $p < 0.0001$, treated 3-IAld vs. untreated virus-exposed cells, One-way ANOVA, Bonferroni post hoc test.
